# End-of-life issues in the acute and critically ill patient

**DOI:** 10.1186/1757-7241-17-21

**Published:** 2009-04-22

**Authors:** Eric A Savory, Catherine A Marco

**Affiliations:** 1 University of Toledo College of Medicine, Mail Stop 1114, 3045 Arlington Avenue, Toledo, Ohio 43614, USA; 2 Professor, Department of Surgery, Emergency Medicine, Director of Medical Ethics Curriculum, University of Toledo College of Medicine, Mail Stop 1114, 3045 Arlington Avenue, Toledo, Ohio 43614, USA

## Abstract

The challenges of end-of-life care require emergency physicians to utilize a multifaceted and dynamic skill set. Such skills include medical therapies to relieve pain and other symptoms near the end-of-life. Physicians must also demonstrate aptitude in comfort care, communication, cultural competency, and ethical principles. It is imperative that emergency physicians demonstrate a fundamental understanding of end-of-life issues in order to employ the versatile, multidisciplinary approach required to provide the highest quality end-of-life care for patients and their families.

## Ethical issues

Patient autonomy, beneficence, non-maleficence, and stewardship of resources comprise the foundation of ethical guidelines for physicians. In recent decades the medical environment has shifted from a paternalistic role of the physician towards the promotion of respect for patient autonomy. Patient autonomy is a respect for an individual's right of self-rule. It implies that a patient best knows his/her own goals and values relating to medical interventions. In addition, patients have the right to make decisions that may conflict with the recommendations of family members and health care providers[1]. This is often a challenging issue for phycians to deal with as patient autonomy may at times conflict with the physician's desire to prioritize beneficence. A physician's duty in such circumstances should be to ensure that the patient is fully informed in order for them to be truly autonomous. Information should include the risks, benefits, and alternatives to the proposed intervention. A physician should also disclose to the patient that his/her decision may conflict with what is in their best interest, in terms of their overall health or survival. Physicians should also elicit a patient's reasoning for preferences and attempt to understand their perspectives and opinions. This process may reveal a cultural factor or experience from a previous medical encounter that contributes to patient preferences. It is also important that a patient's goals for care be established as early as possible [2]. This helps to avoid future confusion and provides a framework from which physicians can understand patient decisions. In addition, when discussing patient preferences about life sustaining treatments, physicians should accurately explain both the possible benefits and burdens to patients[3]. This is important as patients may have preconceived notions of therapies. Their beliefs may be solely based, for example, on a specific publicized case in which the complication or outcome was not necessarily common. Such open communication ultimately allows a patient to make an informed decision about their medical care.

Another important ethical principle is the appropriate stewardship of resources. This is especially significant in end-of-life care when life sustaining treatments can be costly and time consuming. Physicians have a responsibility to avoid letting likely non-beneficial care negatively affect the treatment of other patients. Distributive justice is especially important as technological advances continually allow for new and improved methods of diagnosis and medical treatment. These advances are often expensive, however, and should correspondingly be used only in appropriate circumstances. It should also be added that physicians should avoid taking a passive approach to treatments and procedures with the belief that dying is a non-interventional process. Decisions regarding allocation of resources should not be made at the bedside for individual patients. Individual patient treatment plans should be based on patient preferences, and unbiased evidence regarding outcomes. When discussing outcomes, limitations in evidence to predict individual patient outcomes should be recognized[4,5]. Physicians should advocate for policy and regulatory mechanisms to address the appropriate allocation of resources at the end-of-life.

## The physician's role at the end of life

Emergency physicians face numerous challenges when managing the clinical care of patients at the end-of-life. When appropriate, the goals of emergency medical care are to preserve life. However, patients have the right to choose the goals and objectives of their own medical care. Some patients may choose to forego life-sustaining medical therapies and interventions near the end-of-life.

The primary role of the emergency physician near the end-of-life is to coordinate and administer appropriate medical and psychosocial care for the patient. A statement by the American College of Emergency Physicians states "Emergency physicians should respect the dying patient's needs for care, comfort, and compassion" [6]. (Table 1)

**Table 1:** Ethical Issues at the End-of-life*

To enhance EOL care in the Emergency Department, the American College of Emergency Physicians believes that emergency physicians should:
Respect the dying patient's needs for care, comfort, and compassion.

Communicate promptly and appropriately with patients and their families about EOL care choices, avoiding medical jargon.

Elicit the patient's goals for care before initiating treatment, recognizing that EOL care includes a broad range of therapeutic and palliative options.

Respect the wishes of dying patients including those expressed in advance directives. Assist surrogates to make EOL care choices for patients who lack decisionmaking capacity, based on the patient's own preferences, values, and goals.

Encourage the presence of family and friends at the patient's bedside near the end-of-life, if desired by the patient.

Protect the privacy of patients and families near the end of life.

Promote liaisons with individuals and organizations in order to help patients and families honor EOL cultural and religious traditions.

Develop skill at communicating sensitive information, including poor prognoses and the death of a loved one.

Comply with institutional policies regarding recovery of organs for transplantation.

Obtain informed consent from surrogates for postmortem procedures.

*Excerpted from: American College of Emergency Physicians: Ethical Issues at the End-of-life; Ann Emerg Med 2008; 52:592.

Another important duty of the emergency physician is to educate and counsel the patient and family in order to facilitate decision making[7]. Ultimately, the patient has the final say in decision making, however, a collaborative approach involving the patient, family, and health care team may prove most efficacious. Physicians must gauge patient and family wishes and perspectives, and take on the appropriate level of involvement[8,9]. One of the unique challenges of the emergency physician is to balance these numerous responsibilities in the emergency department environment. The chaotic and fast-paced setting is not always conducive to end-of-life planning, communication, and ensuring patient comfort. Special skills and attention may be required to overcome these obstacles to ensure the most favorable conditions for a patient near the end-of-life.

## Hospice and palliative care

Hospice and palliative care offers many services to patients and their families in the transition towards death. They function to preserve quality of life and pain relief. In addition, Hospice offers support to target the psychological stresses at the end-of-life[10]. Hospice and palliative services are available at acute and chronic care facilities, the patient's home, as well as in the hospital[11]. The primary role of the emergency physician regarding Hospice care should be to educate patients and inform them of its availability. In order for patients and their families to fully utilize Hospice resources, conversations should be raised early in the course of a terminal disease[12]. The goals of the conversation should be to accurately describe Hospice services as well as its availability. Many patients erroneously assume it is expensive, when in fact it has been covered under Medicare since 1983[13]. International advances in palliative care have identified key priorities in palliative care, including financial support, professional training, research, prioritizing pain control, and global awareness of palliative medicine[14-16].

Palliative care is becoming increasingly valued in the age of modern medicine where technological advances are prolonging life and extending survival. Despite recent advances, strides in aspects of palliative care must continue to be made. Many residents still feel that training in the area of pain management is inadequate[17]. While the extension of life is generally considered a positive outcome, it can easily be nullified if it lacks quality. Certainly, its very essence of providing comfort of symptoms is markedly different from some physicians' usual goal of disease cure. It may be difficult for physicians to transition into this mindset and alter their care accordingly. Its importance should not be overlooked, however, as it may be the most important aspect of the end-of-life experience for many patients. Proper palliative care allows patients to shift focus from their condition to their desires for how they choose to spend the precious time remaining.

It is important to stress, as Smith *et al*. describe, that palliative care does not begin when life-prolonging care ends. Rather, these two approaches can be used concurrently. Smith *et al*. provide the example of cancer patients undergoing pain control at the same time that they are receiving disease-modifying therapy. Physicians must find the appropriate balance between these two models, in order to provide the most effective and individualized care for their patients.

Changes in the implementation of palliative care can also be made in order to improve end-of-life care. For example, Wiese *et al*. suggest the use of a palliative care team for terminally ill patients[18]. Patients and their families should have access to assistance from the team 24 hours a day. Their study found that implementation of the palliative care team reduced the number of emergency calls and unnecessary hospitalizations. This type of approach allows for more complete and individualized care as well as the most efficient use of health care resources. In addition to the palliative care team, modes of identifying patients in need of palliative care have proven useful in linking these patients to proper resources[19]. Methods such as those described above would improve the use of the currently underutilized palliative care services.

Pharmacologic management of symptoms at the end-of-life has been a debated issue. Sedation can be effectively used for refractory symptoms, most commonly dyspnea and agitation, that do not respond to other forms of therapy[20]. Mercadante *et al*. demonstrated successful use of controlled sedation and argued that the goal of medical practice should be to avoid symptom distress at the end-of-life. It is important that emergency physicians recognize that such alternatives such as controlled sedation do exist, and when used appropriately, may serve as an effective component of palliative care. Some opponents argue that the use of pain medications such as opioid analgesics, sedative agents, and other symptom controlling measures may hasten a patient's death. However, many ethicists agree that the principle of the double effect is morally permissible; if the provider's intent is to relieve suffering, an unintended effect of influence on the time of death is ethically, legally, and morally acceptable [21-25].

An integral element of palliative care is advanced care planning in which the patient and their family discuss and finalize their wishes regarding end-of-life care. Ideally this would involve the completion of an advance directive such as a DNR order, living will, or durable power of attorney that can carry out the patient's requests. Again, the role of the emergency physician in this process is often difficult given the usual limited time spent with an individual over the course of disease. Nonetheless, emergency physicians should initiate conversations regarding end-of-life planning whenever possible.

One of the most challenging roles of the emergency physician in palliative care is disease assessment and prognostication. Although accurate prognostication is difficult, the closest prognostication involves an evidence-based medicine approach using recognition of specific clinical markers[26]. This requires the physician to be familiar with disease course and utilization and interpretation of diagnostic tools. In addition, a sense of honesty is necessary when communicating information about a prognosis with patients and their families. In order to act in the best interest of the patient, the physician should disclose prognostic information as objectively as possible while avoiding the tendency of providing false hope.

Providing palliative care and caring for dying patients can take a physical and emotional toll on physicians. Hospitals and institutions should, therefore, provide a dynamic set of resources for physician support. Resources may include counseling, case discussion sessions, and other opportunities where medical professionals can express their concerns[27]. In addition, physicians should develop personal coping strategies that allow them to deal with the emotional rigors of their responsibilities.

Numerous symptoms warrant appropriate management at the end-of-life (Table 2) [31].

**Table 2:** Common Symptoms at the End-of-life

Pain
Dyspnea

Anxiety

Depression

Guilt

Nausea

Constipation

Insomnia

Weakness

Fatigue

Delirium

## Pain control

One of the most common debilitating symptoms at the end-of-life is pain. Pain control is an integral element of palliative care. Because pain is a subjective sensation, some health care providers may not adequately recognize and treat pain. Because of this, physicians should devote adequate attention to pain assessment, as well as to the history and physical exam in which the type of pain may be identified. Such factors may aid in medication selection and treatment decisions[28]. Pain assessment scales such as the visual analog scale or three-component scale of mild, moderate, and severe are frequently used. Pain treatment in palliative care should involve regularly scheduled doses with rescue doses available for breakthrough pain. Additionally, pain should be regarded as an individualized symptom unique to each specific individual. Physicians should avoid categorizing patients with similar disease presentations and developing pain treatment protocols for groups of patients[29]. Each patient possesses a unique set of symptoms and pain threshold and should be treated accordingly. Some patients may require very high doses of analgesia to obtain adequate pain relief.

## Dyspnea

Dyspnea is another common symptom at the end-of-life. It is particularly common in the elderly, who may have impaired respiratory function[30]. Dyspnea may be a particularly frightening symptom for patients and families to deal with as it can lead to a sense of panic and anxiety. Dyspnea is often under-treated, with one study finding that 23% of patients experiencing dyspnea in the last 48 hours of life received no documented treatment measures[31]. Current treatment recommendations for dyspnea include opioids, anxiolytics, and oxygen therapy[32].

## Depression and dementia

Depression is a common symptom of the elderly, especially those nearing the end-of-life. It is often unrecognized in the older population for a variety of reasons. First of all, as Emanuel points out, many doctors minimize depression as being a natural reaction in the terminally ill or dying patient[33]. Unfortunately, this type of attitude may lead to under-treatment of depression, further complicating and worsening the dying process. In addition, depression may go undiagnosed as many of the symptoms, such as difficulty sleeping, decreased appetite, or weight loss could potentially be explained by medical causes[34]. Furthermore, there are currently no biological markers or specific diagnostic tests for depression[35]. The diagnosis is primarily based on the psychiatric interview and information that the patient provides. It is often difficult to develop strong patient relationships in the acute care setting of the emergency department. Nonetheless, physicians should keep these considerations in mind and strive to take steps to enhance their relationships with patients. Specific instruments that may be used to quantify depression include the Hospital Anxiety and Depression Scale, visual analog scale, or asking "Are you depressed?" [36-38].

The care of patients with dementia will become an increasingly significant issue as the elderly population continues to grow, especially with the aging baby-boom generation. Dementia poses multiple challenges for emergency physicians providing end-of-life care. In particular, patients with dementia are at an increased risk for infection, such as pneumonia[39]. As Volicer points out, this requires physicians to weigh the benefits of medical interventions against burden to the patient[40]. Such decision making should be a joint collaboration between the health care team, patient, and family. Unfortunately, dementia severely hinders the ability of the patient to express their comfort level, emotions, and wishes regarding medical treatment. Communication with the family is essential when dealing with patients with dementia. Volicer suggests a family conference involving members of the family and health care team[40]. This allows the opportunity for the family to ask questions as well as express their wishes in regard to treatment goals and preferences. Cultural issues may be apparent and it is important for physicians to address and consider the role that such values and traditions will play in patient care[34].

## Cultural and spiritual issues

Cultural issues in health care, particularly at the end-of-life, are becoming increasingly significant as the United States continues to become more diverse. In parallel with this trend, cultural diversity training has found its way into medical school curriculum and other forums. End-of-life decision making and utilization of health care resources should reflect cultural standards and beliefs. Awareness of cultural beliefs, attitudes, and traditions can be important; however, care should be taken not to generalize that all members of a given culture internalize those attributes. Individual assessments and personal communications are imperative to the understanding of cultural influence in any setting. For example, patient autonomy is stressed and of primary importance in the American and much of the European medical environment[41]. However, other cultures, particularly Hispanic and Korean, deem decision making as the responsibility of the family[42]. This diminished sense of patient autonomy may also be reflected in lower advance directive completion rates in some cultures. Many Hispanics, for example, feel that they should not have control over life's processes and thus are less likely to participate in advance care planning[43].

Different patient populations demonstrate varying attitudes that are often manifested in the medical encounter with their physician. Japanese patients often take a reserved approach and may be reluctant to share personal information or feelings with physicians[44]. In contrast to the Japanese, Indian patients tend to be more open and develop a deeper interaction with their physicians. They value the advice of their doctors and correspondingly follow their instructions with medication administration, for example. These attitudes should be taken into account by emergency physicians when informing patients of unpleasant news or attempting to elicit personal feelings in end-of-life planning and decision making.

One particular area that varies among cultures is the communication of bad news. In the United States health care system a physician generally fully discloses the patient's condition no matter how severe it is. In Hispanic and Chinese cultures, however, family members may try to protect loved ones from understanding their condition in full. It is important that emergency physicians remember that they have an ethical and legal obligation to fully inform a patient of their condition. They need to be aware of the fact that family members may attempt to withhold information from the patient. This is particularly pertinent when using a translator. Physicians should use translators that are not related to the patient and request that the information be translated word for word. In addition, physicians should explain to the patients and their families that it is in their best interest to have all the information about their condition so that they can make appropriate decisions and carry out their treatment preferences. However, if a decisional patient elects to forego certain information, that request should be honored.

It is essential to reiterate that cultural competency does not involve memorizing a list of attributes about certain cultures and then applying them to all members of the culture[45,46]. Clearly, this could lead to stereotyping and may impair individualized treatment. Rather, each patient should be treated as a unique individual. Physicians need to recognize that cultural variation does exist and take measures to understand each of their patients' cultural values. The physician should open up a dialogue and invite the patient's perspective on how he/she views and understands illness as well as goals for therapy.

Spiritual concerns play an important role at the end-of-life for many patients. The definition and scope of the term "spirituality" is highly variable. A recent review identified 11 dimensions for end-of-life spirituality, including meaning and purpose in life, self-transcendence, transcendence with a higher being, feelings of communion and mutuality, beliefs and faith, hope, attitude toward death, appreciation of life, reflection upon fundamental values, developmental nature of spirituality, and its conscious aspect[47]. One recent study demonstrated higher use of intensive life-prolonging medical care near death among patients with high reported levels of positive religious coping[48]. Attention to religious issues important to each individual patient and family can be important in ensuring a meaningful end-of-life experience. Most clinicians believe that the provision of spiritual care at the end-of-life should be viewed as a fluid and flexible interpersonal process between health care providers, patients, and families, rather than a set of prescribed rules[49]. Communication regarding religion, belief in God, desire to pray or participate in other religious observances, and desire to involve pastoral care services can be helpful in assisting the patient with meeting spiritual goals at the end-of-life.

## Advance care planning

An advance directive is a legal document completed by a patient enabling their treatment wishes to be carried out if they are unable to make their preferences known. The most common forms of advance directives are the DNR order, living will and durable power of attorney for health care. In the United States, many individual states have legislation to recognize state-approved DNR orders and identification. A living will is a record declaring a patient's provisions for their care. Common misconceptions of living wills include the belief that they are only useful for elderly patients and that a lawyer is required to complete one.

A durable power of attorney for health care is when a patient proactively assigns a surrogate decision maker in the event that they are incapable of making medical decisions themselves. Although the durable power of attorney is most commonly a spouse or other family member, a trusted friend can also fulfill the duty. It is imperative that individuals openly discuss their end-of-life preferences with their surrogate decision maker in order to fully utilize the purpose of this form of advance directive.

Advance directives serve many important functions, primarily the communication of individual patient wishes regarding end-of-life care. Many individuals have strong personal preferences regarding cardiopulmonary resuscitation[50]. These preferences vary widely, and are dependent on a variety of factors, including age, state of health, and clinical setting[51,52]. Recent reports suggest that full resuscitative efforts are not necessarily desired by most patients, and that trends toward societal consensus in hypothetical resuscitation scenarios can be identified[53,54]. Without advance directives, providers and families often erroneously judge the patient's end-of-life wishes [55-59].

The Patient Self-Determination Act, passed by the U.S. Congress in 1990, requires Medicare and Medicaid providers to inform patients of their rights regarding advance directives[60]. Its intent was to generate public awareness regarding patient care and rights at the end-of-life. Unfortunately, however, the policy has failed to fulfill expectations regarding an improvement in public knowledge and advance directive completion rate [61-64]. In addition, studies have found that individuals that have advance directives do not uniformly understand their implications[61,62,65]. This general lack of public understanding of end-of-life issues serves to further complicate the role of the physician and outcomes for the patient. Additionally, results of previous studies have demonstrated that physicians overestimate their patients' health literacy levels [66-68]. Improvement of health literacy among patients should enhance autonomy and facilitate discussions with their physicians. Regardless of the level of public understanding on these issues, it is the physician's responsibility to generate dialogue and inform individuals of their rights as patients. Even brief educational interventions can prove to be beneficial in improving patient understanding[69].

Only a minority of patients (estimated 10–30%) have completed advance directives [70-74]. and even fewer present to the ED with the appropriate documentation[75]. Several studies indicated that individuals who have completed advance directives do not fully understand their implications[61,62]. Although an important concept, one of the biggest challenges in the widespread implementation of advance directives is appropriate communication and implementation[76,77].

The ideal advance directive allows a system to honor directives that are comprehensive, preserve patient autonomy, and can be easily understood by everyone involved[78]. One example of such a system is the POLST initiative, which has been successful in Oregon in furthering the cause of patient-directed end-of-life planning[79].

## Communication with patients and family members

Communication is a key aspect of end-of-life care. Many even rank communication skills as having equal or greater importance than clinical skills[80]. It is one of the most important responsibilities of physicians, particularly near the end-of-life when patients and families are most vulnerable. Proper communication is essential throughout the entire disease course as a patient's goals and preferences may change over time, due to a multitude of factors[81]. There are several communication techniques that should be routinely practiced (Table 3) [82-85]. The use of open-ended questions towards patients and their families allows physicians to assess baseline knowledge about the particular situation[82,83]. Von Gunten, *et al*. also recommends frequent pauses in conversation, especially after transmission of bad news. This allows for patients to integrate the information as well as physician interpretation of patient understanding. Other important communication techniques may include eye contact, speaking from a seated position, empathy, and reflective listening.

**Table 3:** Effective Communication Tips at the End-of-life

Ensure a quiet location for communication
Allow appropriate time for the interaction

Involve the family in all communication

Involve the family in all communication

Speak from a seated position

Assess patient understanding

Use open ended questions to gauge understanding and invite the patient perspective

Accept and address emotions of patients and loved ones

Educate patient and family on disease state

Inquire about and address cultural issues

Demonstrate reflective listening

Express empathy

Disclose all benefits and risks to treatment as well as alternative therapy

Speak objectively in an evidence-based manner

Suggest all appropriate resources to patients and their families including psychiatric care, pastoral support, and social services

Identify and discuss advance directives

Establish patient goals and develop a plan

## Informed consent and decision-making capacity

Informed consent is a fundamental patient right that serves to protect patient autonomy regarding treatment options. Consent to medical treatment is not required in limited extraordinary circumstances. Emergency treatment is one such exception. It is important, however, that emergency physicians not abuse this exception. In most clinical circumstances, informed consent should be obtained from the patient, and if he/she is not capable, from the family or surrogate[84]. This requires that emergency physicians adequately assess situations to determine the urgency level and whether or not informed consent can feasibly be obtained.

Informed consent requires three elements: decisional capacity, delivery of information, and voluntariness. In order for informed consent to be obtained, the patient must demonstrate decision making capacity. No simple, universally accepted capacity assessment exists, as determination of capacity involves a number of variables, including patient ability to receive information, deliberate, and communicate their choices[87]. Capacity can be affected by numerous clinical conditions, including pain, anxiety, depression, delirium, intoxication, medication effects, and numerous others[86-88]. Some standardized tests such as the mini-mental status exam (MMSE) can be used to evaluate patient orientation or memory, but do not necessarily assess patient understanding of the risks versus benefits of treatment options, for example[88,89]. Physicians should utilize a systematic algorithm when assessing patient capacity[89]. Miller and Marin's proposed algorithm, for example, involves a stepwise approach that evaluates patient communication about a choice as well as understanding of informed consent and the risks and benefits of the medical intervention[87]. Following a validated systematic process minimizes physician bias and standardizes the measurement of capacity in each individual situation.

## Euthanasia and physician-assisted suicide

Euthanasia and physician-assisted suicide are emotionally charged and debated topics in American medicine. Euthanasia has been defined as the intentional ending of the life of a person suffering from an incurable or terminal illness[90]. Physician-Assisted Suicide has been defined as a practice in which the physician provides a patient with a lethal dose of medication, upon the patient's request, which the patient intends to use to end his or her own life[91]. These controversial practices have found their way into the public arena due in large part to the legalization of physician-assisted suicide in Oregon in 1997 as well as the publicity attributed to Dr. Jack Kevorkian in the 1990's. Despite such exposure, there may exist a failure of distinction between euthanasia and physician-assisted suicide[62,92]. Currently, in the United States, physician-assisted suicide is legal only in the states of Oregon and most recently Washington, and euthanasia is illegal in every state[93]. Euthanasia is, however, legal in some countries, including the Netherlands and Switzerland. Interestingly, many individuals support widespread legalization of both euthanasia and physician-assisted suicide[93]. Despite this support it is essential that emergency physicians follow the laws of the state in which they practice. Physicians must explain their legal obligations even if patients express wishes regarding one of these forms of death. When treating patients who make such requests, physicians should attempt to understand the root of these feelings. Many patients may contemplate physician-assisted suicide out of fear that could potentially be resolved with education about their disease and effective palliative symptom management.

## Conclusion

Emergency physicians play a multifaceted role in the end-of-life care of the acute and critically ill patient. The responsibilities extend far beyond the essential intellectual and clinical skills. The physician must also possess competency in communication, empathy, cultural, and ethical issues. Complete care involves an integration of all of these factors, resulting in a multidimensional, patient-specific approach.

## Future directions and closing remarks

Future efforts should focus on training emergency physicians in the appropriate end-of-life care. Advocacy and education should be instituted at all levels. Emergency departments should establish policies and procedures to support the ethical and compassionate provision of end-of-life care. In addition, medical schools and GME training programs should continue to incorporate cultural competency and patient-centered training into the curriculum. Such abilities are not innate, but rather, they can and should be continually refined and improved. In the context of these matters, physicians should take time to reflect on their overall goals and purpose of their practice in order to fully utilize their skills in the improvement of the human condition near the end-of-life.

## Competing interests

The authors declare that they have no competing interests.

## Authors' contributions

ES and CAM drafted and revised the manuscript. All authors read and approved the final manuscript.
